# Treating Cancer by Targeting Telomeres and Telomerase

**DOI:** 10.3390/antiox6010015

**Published:** 2017-02-19

**Authors:** Marko Ivancich, Zachary Schrank, Luke Wojdyla, Brandon Leviskas, Adijan Kuckovic, Ankita Sanjali, Neelu Puri

**Affiliations:** Department of Biomedical Sciences, College of Medicine at Rockford, University of Illinois, Rockford, IL 61107, USA; msi4@students.calvin.edu (M.I.); zacharyschrank15@augustana.edu (Z.S.); lukewojdyla@gmail.com (L.W.); brandonleviskas@gmail.com (B.L.); ak147356@rockford.edu (A.K.); ankita.sanjaali@gmail.com (A.S.)

**Keywords:** telomerase, DNA damage responses, G-quadruplex, guanine-rich oligonucleotides (GROs), telomere homolog oligonucleotides (T-oligos), shelterin complex

## Abstract

Telomerase is expressed in more than 85% of cancer cells. Tumor cells with metastatic potential may have a high telomerase activity, allowing cells to escape from the inhibition of cell proliferation due to shortened telomeres. Human telomerase primarily consists of two main components: hTERT, a catalytic subunit, and hTR, an RNA template whose sequence is complimentary to the telomeric 5′-dTTAGGG-3′ repeat. In humans, telomerase activity is typically restricted to renewing tissues, such as germ cells and stem cells, and is generally absent in normal cells. While hTR is constitutively expressed in most tissue types, hTERT expression levels are low enough that telomere length cannot be maintained, which sets a proliferative lifespan on normal cells. However, in the majority of cancers, telomerase maintains stable telomere length, thereby conferring cell immortality. Levels of hTERT mRNA are directly related to telomerase activity, thereby making it a more suitable therapeutic target than hTR. Recent data suggests that stabilization of telomeric G-quadruplexes may act to indirectly inhibit telomerase action by blocking hTR binding. Telomeric DNA has the propensity to spontaneously form intramolecular G-quadruplexes, four-stranded DNA secondary structures that are stabilized by the stacking of guanine residues in a planar arrangement. The functional roles of telomeric G-quadruplexes are not completely understood, but recent evidence suggests that they can stall the replication fork during DNA synthesis and inhibit telomere replication by preventing telomerase and related proteins from binding to the telomere. Long-term treatment with G-quadruplex stabilizers induces a gradual reduction in the length of the G-rich 3’ end of the telomere without a reduction of the total telomere length, suggesting that telomerase activity is inhibited. However, inhibition of telomerase, either directly or indirectly, has shown only moderate success in cancer patients. Another promising approach of targeting the telomere is the use of guanine-rich oligonucleotides (GROs) homologous to the 3’ telomere overhang sequence (T-oligos). T-oligos, particularly a specific 11-base oligonucleotide (5’-dGTTAGGGTTAG-3’) called T11, have been shown to induce DNA damage responses (DDRs) such as senescence, apoptosis, and cell cycle arrest in numerous cancer cell types with minimal or no cytostatic effects in normal, non-transformed cells. As a result, T-oligos and other GROs are being investigated as prospective anticancer therapeutics. Interestingly, the DDRs induced by T-oligos in cancer cells are similar to the effects seen after progressive telomere degradation in normal cells. The loss of telomeres is an important tumor suppressor mechanism that is commonly absent in transformed malignant cells, and hence, T-oligos have garnered significant interest as a novel strategy to combat cancer. However, little is known about their mechanism of action. In this review, we discuss the current understanding of how T-oligos exert their antiproliferative effects in cancer cells and their role in inhibition of telomerase. We also discuss the current understanding of telomerase in cancer and various therapeutic targets related to the telomeres and telomerase.

## 1. Introduction

**Figure 1:**
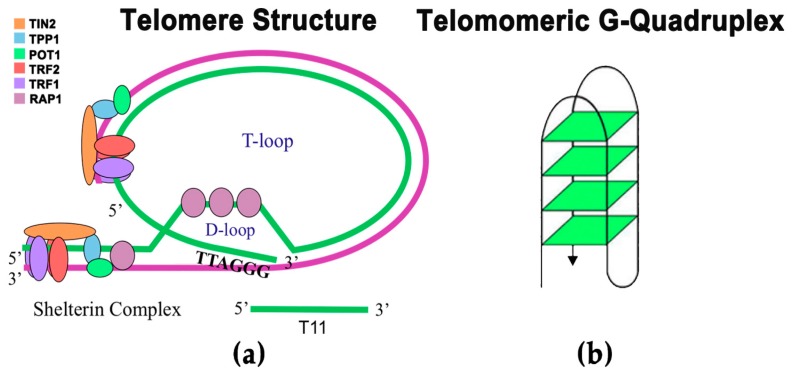
(**a**) Telomeric DNA has the ability to fold over itself, forming what is called the T-loop. Furthermore, the 3’ single-stranded overhang can tuck under itself, forming what is called the D-loop. Additionally, there are a group of regulatory proteins attached to the telomere at several locations, called the shelterin complex, which maintain telomere homeostasis; (**b**) Telomeric DNA forms intramolecular G-quadruplexes.

Mammalian chromosome ends are capped by protective DNA structures and DNA-binding proteins, collectively known as telomeres [1]. In adult somatic cells, telomeres are composed of large noncoding sequences of approximately 1000–2000 TTAGGG tandem base pair repeats that end in a 3′ extension past the 5′ terminus [2]. In other select cells, the telomeres are maintained and can be considerably longer, such as adult human germ cells which can contain anywhere from 500–5000 repeats [3,4,5]. During each cycle of cell division telomeres are incompletely replicated, and consequently, their ends are progressively shortened [6]. When these telomeres reach a critically short length, DNA damage responses (DDRs) such as apoptosis and cellular senescence are induced. Hence, telomeres are considered to be “biological clocks”, as they limit the proliferative potential of most normal cells [2,7]. It has been demonstrated that telomeres exist in a secondary structure known as the t-loop, which is formed by the invasion of the 3’ overhang into the duplex region of the telomere and is stabilized by the shelterin complex, a grouping of six proteins that regulate telomeric stability and homeostasis (Figure 1) [2,8,9]. The shelterin complex is composed of the proteins TRF1, TRF2, POT1, TIN2, TPP1, and Rap1, which bind to the single-stranded and/or double-stranded regions of the telomere (Figure 1) [2,9,10]. The shelterin complex also plays an integral role in the regulation of telomerase and the prevention of telomere degradation by nucleases. When TRF1, TRF2, or POT1 are not functioning properly or dissociate from the telomere, the t-loop unfolds, exposing the telomere, which induces DDRs such as apoptosis and senescence [1,11,12]. It is thought that the T-loop is also stabilized by the guanine-rich (G-rich) character present in telomeres [6,12,13], allowing the telomere 3’ overhang to take on a G-quadruplex structure, a secondary structure formed from the hydrogen bonding of guanine residues in tetrad formations (Figure 1). These G-quadruplexes stack together at the D-loop, the area where the 3’ end of the telomere penetrates the duplex region [2,10]. It is thought that these G-quadruplexes have a crucial role in the blocking of the enzyme telomerase from gaining access to the telomere [1,12].

Telomerase is inappropriately expressed in roughly 85%of cancers, and the level of its activity is higher in advanced and metastatic tumors, making it a viable cancer biomarker and therapeutic target. While germ line cells, stem cells, and select others including cardiovascular cells do express detectable levels of telomerase, it is mostly quiescent, and most normal cells have minimal or no detectable telomerase activity [14,15,16,17]. Telomerase confers cellular immortality through the addition of TTAGGG tandem repeats to the 3’ end of the telomere, thereby maintaining stable telomere length, preventing the induction of DDRs by the critical shortening of telomeres, and allowing continued proliferation of neoplastic cells [7]. Telomerase is comprised of two main components: hTERT, the catalytic subunit of telomerase which catalyzes the addition of nucleotides to the 3′ overhang; and hTR, an RNA template complementary to the 3′ overhang which acts as a primer, in addition to telomerase-associated proteins hTEP1, p23, Hsp90 and dyskerin [10,18,19]. Limiting the growth potential of tumors has been the focus of chemotherapeutic intervention for decades, and because of their selective expression in neoplastic growths, telomerase and telomeres have become attractive and novel targets for the development of anticancer therapeutics [2,10].

A minority of cancers, less than 15%, maintain telomere length homeostasis through the alternative lengthening of the telomeres (ALT) pathway. ALT-positive cells are able to replenish telomeric DNA in a telomerase-independent manner through a homologous recombination-mediated replication mechanism, and are thus resistant to telomerase-based therapies [1,11]. However, the mechanism of ALT is still poorly understood, and it is possible that multiple mechanisms of ALT exist [20]. In addition, it is thought that resistance to telomerase inhibitor therapy may occur through the activation of ALT pathways in some cancers [1].

## 2. Current Therapies Related to Telomeres and Telomerase

Due to its over-expression in the majority of cancers, and minimal or nonexistent expression in most somatic cells, telomerase is a unique cancer biomarker. Thus, telomerase and other telomere components are attractive targets for developing therapeutics. Many telomere-based therapies are currently under investigation, including telomerase inhibitors, tankyrase inhibitors, and guanine-rich oligonucleotides (GROs) [2,10].

Currently there is a multitude of drugs designed to directly inhibit telomerase. GRN163L, also called Imetelstat, has been one of the most widely developed and is arguably the most successful [21]. GRN163L is a 13-mer oligonucleotide that acts as a direct telomerase inhibitor by antagonistically binding to the RNA template of telomerase (hTR) [21,22]. Preclinical studies utilizing GRN163L have shown effective inhibition of telomerase. Treatment on breast cancer cells demonstrated a reduction in cell tumorigenicity and invasiveness [23,24]. However, the effects of long-term treatment with telomerase inhibitors have not yet been investigated and there is currently no clear information on their effects on normal cells that transiently express telomerase, such as germ cells. Studies by Herbert et al. with modified oligonucleotides as telomerase inhibitors, using immortalized breast cancer cells, indicate that telomere shortening is reversible and may have limited side effects on stem cells [25]. Furthermore, several cancers maintain very short telomeres that are maintained by telomerase, and evidence suggests that increased tumorigenicity occurs due to dysfunction of these telomeres [1,19,26]. These shortened telomeres of tumors may erode to a critical length before irreversible harm occurs to telomerase-positive stem cells [25].

Indirect inhibition of telomerase can occur via the silencing of tankyrase 1 (TNKS1), a protein that PARsylates TRF1 during the S-phase of cell division and displaces it from the telomere, which is required for telomerase activity [27,28,29]. Depletion of TNKS1 with siRNA knockdown results in telomere uncapping and increased sensitivity to ionizing radiation, which could in turn be potentially useful to cancer patients [30]. shRNA against tankyrase 1 has been shown to reduce cell viability in cancers [31]. Furthermore, studies have demonstrated enhanced telomerase inhibition through the inhibition of tankyrase 1 in conjunction with telomerase inhibitors such as MST-312 [31,32]. Combinatorial therapy using tankyrase 1 inhibitors and telomerase inhibitors may have an even greater inhibitory effect on telomerase and thus may be an effective anticancer therapeutic.

Another strategy aimed at overcoming telomerase-related immortality is the increased stabilization and formation of endogenous telomeric G-quadruplex secondary structures within the telomere. G-quadruplexes (G4) form naturally in telomeric regions and it is thought that the steric hindrance at these regions may disrupt telomerase activity in these regions [33]. The stabilization of G-quadruplexes can prevent telomerase from accessing and elongating the telomere, thus impeding the progression of the replication fork through telomeric tracts [1,2,34]. However, telomerase has no role in resolving G4-structures along the telomeric repeats since they are resolved by the DNA helicases [34]. As such, the use of G-quadruplex–stabilizing ligands has potential applications for the development of treatments for malignant and progressive cancers. These agents bind with high affinity to the 3’ single-stranded region of the telomere, facilitating formation and stabilization of the G-quadruplexes. Some of the most promising G-quadruplex–stabilizing ligands include telomestatin, BRACO-19, and RHPS4, and studies utilizing these agents have demonstrated increased G-quadruplex stability and upregulation of DDRs in cancer cells [33,35,36,37,38]. Long-term treatment with these agents has also demonstrated a reduction in 3’ overhang length without a reduction in the overall telomere length, which suggests the inhibition of telomerase [39]. 

Recent studies have suggested that the reverse transcriptase catalytic subunit (TERT) of telomerase mediates other oncogenic activity in addition to its role in telomere elongation [40,41]. TERT upregulation or reactivation seems to be associated with several “hallmarks of cancer” including increased mitochondrial activity, DDR signaling, and WNT/β-catenin signaling [42,43,44]. More recently, it was shown that TERT regulates MYC-driven oncogenesis independently of its reverse transcriptase activity [13]. TERT-null mice showed a delayed development of MYC-induced lymphomagenesis, while Terc-null mice did not [45]. Studies after ectopic expression of TERT showed enhanced translation in cells to regulate cancer cell proliferation independent of telomere length, suggesting that the effects of TERT could be telomere-independent [46]. Additionally, it has been known that TERT is a major component and limiting factor for telomerase activity [41,47]. Based on these findings, we suggest that a human TERT (hTERT) inhibitor may be a more efficient therapeutic than current hTR inhibitors since, it would prohibit telomere elongation in addition to halting TERT’s non-canonical effects.

TERT expression is often increased in cancer due to point mutations in TERT’s promoter region, resulting in the recruitment of multiple different potential transcriptions factors that upregulate TERT and thus increase telomerase activity [48,49,50,51]. TERT expression has been demonstrated to be dependent on MAPK pathway activation which is mediated by transcription factors of the ETS (E26 transformation-specific) family in melanoma cells [51]. Examination of TERT reactivation through the recruitment of ETS transcription factors demonstrated that the process is dependent on non-canonical NF-κB signaling [48]. Given TERT’s potential role in multiple oncogenic proliferative processes and its association with ETS, future studies that target inhibition of mutant TERT promoters appear to be a promising strategy.

Current therapeutic agents that promote telomere attrition through direct or indirect inhibition of telomerase have demonstrated limited improvement in cancer patient prognosis [13,21]. Although the inhibition of telomerase may strip some cancers of their immortality, cancers are still viable and largely unaffected by the loss of telomerase. Furthermore, some cancers develop resistance to these inhibitors by means of ALT, rendering them totally ineffective [52]. Targeting hTERT, specifically via the inhibition of TERT promoter- or mutant promoter-mediated transcription, may be a far more effective strategy [49,53]. Additionally, a potentially promising telomere-related therapy is the use of guanine-rich oligonucleotides (GROs), specifically those that are homologous to the telomere, to disrupt the telomere [1].

## 3. Telomere Homolog Oligonucleotides

Commonly known as T-oligos, telomere homolog oligonucleotides are known to have potent anticancer effects when administered to several malignant cell types, both in vitro and in vivo. Our lab has studied a number of these oligonucleotides in several different cancer cell lines, and our current research centers around one particular 11-base oligonucleotide (5′-dGTTAGGGTTAG-3′) called T11 [1,2]. T-oligos accumulate in the nucleus and rapidly induce DDRs mediated by ATM, p53, E2F1, cdk2, and p95/NBS1 and their downstream targets, resulting in cell cycle arrest, senescence, apoptosis, and differentiation [54,55,56,57,58].

In vitro studies demonstrate that T11 is highly effective in reducing viability and growth of several cancers including melanoma, lung, prostate, ovarian, breast, and colorectal [1,2,56,57,58,59,60,61,62,63]. Treatment with T11 demonstrated upregulation of several tumor differentiation markers in colorectal cancer, which have roles in inhibiting proliferation and are lost in poorly differentiated cancers [61]. T11 treatment also induces the upregulation of several melanoma differentiation proteins which are currently the targets of melanoma vaccine therapies [63]. Combination treatment of T11 with a tyrosine kinase inhibitor or histone deacetylase inhibitor that are currently used clinically, have demonstrated additive inhibition of cancer cell cellular growth [61,62]. Furthermore, when combined with ionizing radiation treatment, T11 increased radiosensitivity and synergistically inhibited cell and tumor growth both in vitro and in vivo, respectively [59].

T11 also stimulates various anti-cancer responses in vivo, such as reduction of tumor burden and metastatic potential in mice, with no detectable toxicity [63,64,65,66,67]. Our lab has shown that tumor volume in mice with NSCLC H358 and SW1573 lung cancer xenografts was reduced by 80% and 88%, respectively, after treatment with T11 for seven weeks [64]. Another in vivo study on melanoma reported that, when compared with controls, T11 reduced the average number of metastases by 90%–95% and tumor volume by 84%–88% [63]. T11 also demonstrated its ability to elicit its anti-tumor effects by inducing senescence and inhibiting angiogenesis in melanoma and lung cancer [63,64,65].

Although the DDRs initiated by T11 have been intensely studied and are mostly elucidated, the process by which T11 initially activates these DDRs is not completely understood [1,63,68]. It has been proposed that T11 mimics the physiological signal of telomere exposure [57,63], since it elicits DDRs similar to those induced by telomere dysfunction after ectopic expression of a non-functional TRF2 [69,70]. Studies have shown that T11 upregulates the shelterin protein TRF2 [71]. Recently, preliminary studies from our laboratory showed that TRF2 and POT1 are upregulated after treatment with T11, and immunofluorescence studies performed in our lab showed possible co-localization of these proteins with T11.

**Figure 2:**
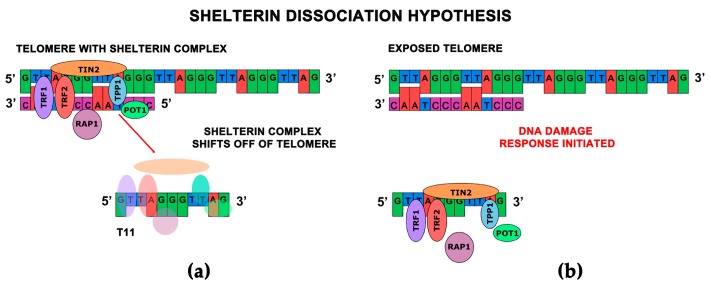
(**a**) The shelterin dissociation model hypothesizes that the presence of T11 in the nucleus causes the disruption of the telomere, particularly the shelterin complex. This model suggests that T11 competes with telomeric DNA for the binding of shelterin proteins; (**b**) Key proteins of the shelterin complex are displaced from the telomere and/or bind to T11. This disruption leaves the telomere exposed and initiates DDRs.

**Figure 3:**
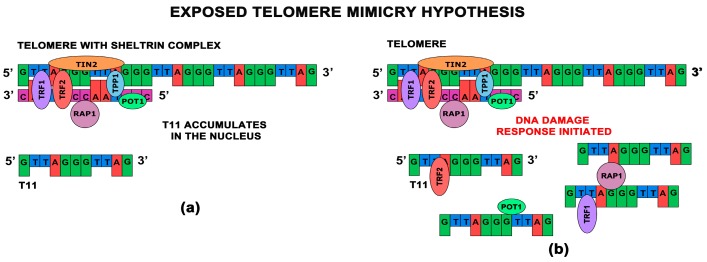
(**a**) The exposed telomere mimicry model hypothesizes that exogenous T11 mimics the exposed endogenous telomere overhang; (**b**) The cell responds to this mimicry of the telomere overhang exposure by initiating DDRs in cancer cells and triggering upregulation of shelterin proteins in attempt to overcome apparent telomere exposure.

From these findings we are able to conclude that there are two potential modes of T-oligo action. We have designated the first mode as the shelterin dissociation model (SDM) and the other we refer to as the exposed telomere mimicry model (ETM). The SDM hypothesizes that the introduction of T11 into the nucleus displaces shelterin proteins from the telomere, thereby critically compromising the telomere secondary structure. It is thought that at least some shelterin proteins bind T11, resulting in the opening of the T-loop causing DDR signaling (Figure 2). The ETM model hypothesizes that T11 accumulates in the nucleus and is recognized as an exposed or damaged telomere, thus initiating DDRs identical to those that occur under normal physiological conditions when telomeres are critically shortened (Figure 3). It is also possible that both potential modes are occurring simultaneously and that T11’s effectiveness is due to both its ability to disrupt the shelterin complex and mimic telomere exposure. In summary, in the exposed telomere mimicry model exogenous T11 mimics the exposed telomere overhang, resulting in initiating DDRs in cancer cells. In the shelterin dissociation model, the presence of T11 in the nucleus causes the disruption of the telomere and the shelterin complex since T11 competes with telomeric DNA for the binding of shelterin proteins, resulting in telomere overhang exposure and initiation of DDRs. Since the activity of telomerase is independent of T11, it will not be modulated in either model [72,73]. Further studies are needed to improve our understanding of these mechanisms.

Interestingly, T11 and similar GROs have been shown to have little or no antiproliferative effect on normal cells studied and had no detectable toxic effects in mice [2,63,67]. It is important to note that T11 and other GROs have only been tested in a number of non-malignant tissues and treatment times vary between three to six days [60,63,64]. Furthermore, murine telomeres are five to 10 times longer and have constitutive expression of telomerase compared to humans, resulting in reduced species longevity in mice [74,75]. Laboratory mice and humans also have differences in the sequence homology of telomeric proteins TRF1 and POT1 [76,77,78]. This indicates that T-oligo–initiated DDRs are specific to cancer cells and may be due to changes in telomere physiology that are common among cancers. Silencing hTERT in oral cancer cells is accompanied by caspase-9/-3 cleavage, DNA damage responses, and decreased cell viability [79]. It may be possible that T11’s antiproliferative response is in some way hTERT-dependent or hTERT-mediated due to its homology to the telomere, and therefore selective to cells that express the telomerase enzyme. Further studies should be done to elucidate the role hTERT plays in T11-induced DDRs in addition to analysis of T11’s effect on non-cancerous telomerase positive cells. The effects of T-oligo on cells that exhibit ALT are also poorly understood, and should be a subject of future studies. However, one study conducted using ALT-positive U20S osteosarcoma cells demonstrated a function for WRN in T-oligo–induced responses [80]. WRN is a protein which has exonuclease and helicase activity and is defective in Werner Syndrome patients [80]. Downregulation of WRN using siRNA decreased phosphorylation of γH2AX, p53 and ATM in T-oligo–treated osteosarcoma cells [80]. Though this study demonstrates that T-oligo induces DNA damage responses in ALT-positive cells, further investigation into this area is a necessity.

Despite its effectiveness, T11 has limited stability in vitro and in vivo due to degradation by nucleases [66]. To enhance its stability and delivery, our lab has recently complexed T11 with a cationic helical polypeptide, PVBLG-8 (PVBLG) [66]. The helical structure of PVBLG allows it to remain stable across a wide range of pH values, temperatures, and salt concentrations in the presence of denaturing agents, making it an excellent delivery vehicle [66,81]. It has been shown to be a highly efficient vehicle for plasmid delivery and siRNA in many cell lines [81,82]. PVBLG is cationic and self-assembles with negatively charged oligonucleotides such as T11, neutralizing their charges and stabilizing them for optimal delivery [66]. When complexed with PVBLG, T11’s cellular uptake was improved on a log scale and its ability to inhibit cellular growth and reduce tumor burden in mice was significantly enhanced [66]. Melanoma tumors grown on the flanks of SCID mice and subsequently treated with T11 in the presence or absence of PVBLG showed a three-fold and nine-fold reduction in tumor size, respectively [66]. However, since PVBLG is currently difficult to synthesize, a more efficient yet equally effective alternative is needed in order for it to be produced on a large scale for cancer treatment.

Novel lipid nano-particles are being researched by our lab as a potential alternative for PVBLG. In theory, lipid nanoparticles will form a liposome around T11, thereby increasing its cellular uptake and minimizing degradation. However, this delivery system has had limited success as many of the current lipids have high degrees of toxicity [83,84]. In addition, it was recently discovered that many guanine-rich oligonucleotides, such as T11, are able to form G-quadruplexes [57]. We suggest that the additional secondary structure of a G-quadruplexed T11 may confer added stability in serum, thus increasing its efficacy which is supported by preliminary studies in our laboratory.

## 4. Conclusions

Molecularly targeted therapies are a cornerstone for the treatment of aggressive cancers [85]. Due to the role that telomeres play in the life cycle of cancers, telomerase and telomere-based therapeutics may be able to replace conventional treatments in the future. Undesirable side effects that conventional therapies elicit may be reduced or eliminated due to the specificity of these treatments. There is a small percentage of cells that express telomerase, such as germ cells and stem cells. Extended exposure to telomerase-targeted therapies may potentially inhibit growth of these stem cells. Normal-tissue stem cells are telomerase-competent. However, telomerase is mostly silent with minimal activation in stem cells, while cancer cells are almost universally telomerase-expressing and active [16,17]. Currently, the effects of telomerase inhibition on stem cells with telomerase activity are unclear and require further study [2].

In this review, we described the two current proposed mechanisms of action of T11 and other related short-stranded telomere-homologous oligonucleotides (Figure 2 and Figure 3). However, further research is needed to investigate both these models. It is also critical to determine why the antiproliferative responses of T11 appear to be limited only to malignant cells [1,2,68], which would aide researchers in progressing this therapy into clinical trials. Additionally, exploring T11’s mechanism of action may lead to the discovery of novel potential targets for future therapies. T11 and other T-oligos show promise in preclinical studies; however, there is a hindrance in their delivery in vivo due to their degradation by serum and intracellular nucleases [66]. Stabilization of oligonucleotides by nanocomplex formation with cationic polypeptides is a potential solution, since they have excellent bioavailability and confer enhanced gene delivery with minimal toxicity [66]. Due to its high activity in the majority of cancers and low expression in somatic cells, telomerase and the telomere as a whole are very promising targets [2,10]. Continued studies on telomeres may provide promising avenues of research in the development of novel anticancer therapeutics. G-quadruplex stabilizers could provide a viable means of blocking telomerase [1]. These therapeutics may prove to be more effective than direct or indirect inhibition of telomerase in decreasing cancer cell survival due to their ability to form telomeric G-quadruplexes in situ. Another novel approach of inhibition of telomerase activity in cancer cells relies on the inhibition of tankyrase-1, which shortens telomeres in cancer cells and initiates DDRs [2]. Studies have demonstrated that the suppression of telomerase activity by these inhibitors can eliminate resistance to telomerase inhibition and may be used in combinatorial clinical therapies [31]. In the future, combination therapies of telomerase inhibitors and standard-of-care or traditional therapies may be the most effective way to target telomerase-positive tumors [2]. These modalities could include combination therapy with targets of the non-canonical variety which can eliminate resistance to telomerase inhibition and may be used in conjunction in clinical therapies [40].
